# Histological Methods to Assess Skeletal Muscle Degeneration and Regeneration in Duchenne Muscular Dystrophy

**DOI:** 10.3390/ijms232416080

**Published:** 2022-12-16

**Authors:** Nicolas Dubuisson, Romain Versele, Chloé Planchon, Camille M. Selvais, Laurence Noel, Michel Abou-Samra, María A. Davis-López de Carrizosa

**Affiliations:** 1 Endocrinology, Diabetes and Nutrition Unit, Institute of Experimental and Clinical Research, Medical Sector, Université Catholique de Louvain (UCLouvain), Avenue Hippocrate 55, 1200 Brussels, Belgium; 2 Neuromuscular Reference Center, Cliniques Universitaires Saint-Luc (CUSL), Avenue Hippocrate 10, 1200 Brussels, Belgium; 3 Departamento de Fisiología, Facultad de Biología, Universidad de Sevilla, 41012 Seville, Spain

**Keywords:** dystrophin, histology, Duchenne muscular dystrophy, skeletal muscle, myofibre, animal model, regeneration, degeneration, immunofluorescence, immunohistology

## Abstract

Duchenne muscular dystrophy (DMD) is a progressive disease caused by the loss of function of the protein dystrophin. This protein contributes to the stabilisation of striated cells during contraction, as it anchors the cytoskeleton with components of the extracellular matrix through the dystrophin-associated protein complex (DAPC). Moreover, absence of the functional protein affects the expression and function of proteins within the DAPC, leading to molecular events responsible for myofibre damage, muscle weakening, disability and, eventually, premature death. Presently, there is no cure for DMD, but different treatments help manage some of the symptoms. Advances in genetic and exon-skipping therapies are the most promising intervention, the safety and efficiency of which are tested in animal models. In addition to in vivo functional tests, ex vivo molecular evaluation aids assess to what extent the therapy has contributed to the regenerative process. In this regard, the later advances in microscopy and image acquisition systems and the current expansion of antibodies for immunohistological evaluation together with the development of different spectrum fluorescent dyes have made histology a crucial tool. Nevertheless, the complexity of the molecular events that take place in dystrophic muscles, together with the rise of a multitude of markers for each of the phases of the process, makes the histological assessment a challenging task. Therefore, here, we summarise and explain the rationale behind different histological techniques used in the literature to assess degeneration and regeneration in the field of dystrophinopathies, focusing especially on those related to DMD.

## 1. Introduction

Duchenne muscular dystrophy (DMD) is a severe X-linked inherited myopathy characterised by the mutation of the dystrophin encoding gene named DMD [1]. Dystrophin is a key scaffolding protein providing structural stability and integrity to muscle fibre membranes. The lack of this protein produces higher susceptibility to injury during contraction, thereby resulting in endless cycles of myofibre necrosis and regeneration, ultimately leading to fibrosis, adipogenesis and weakness [2,3].

Besides dystrophin mutations which represent the primary cause of DMD, persistent inflammation and impaired regeneration are likely to be other events that exacerbate the disease progression [4]. The inflammatory process typically starts with a mechanical stress occurring on dystrophin-deficient fibres. Intracellular Ca^2+^ levels increase first, to the malfunction of a great variety of proteins and channels responsible for the correct storage and release of the ion, the so-called Ca^2+^ toolkit [5], and secondly, as a consequence of the structural breakdown of the sarcolemma [6]. Ca^2+^ toolkit proteins are present not only in the plasma membrane, but also in storing organelles such as the sarcoplasmic reticulum (SR) and, to a lesser extent, the mitochondria. Abnormal Ca^2+^ homeostasis ultimately leads to protein degradation, mitochondrial dysfunction and necrosis [7,8]. The presence of necrotising myofibres will then attract M1 macrophages, among other cells, which are responsible for the clearance of debris and necrotic myofibres. These immune cells secrete proinflammatory cytokines leading to the extension of the inflammatory process [4]. After several rounds of muscle degeneration, inflammation becomes chronic and the continuous process of satellite cell (SC) activation exhausts the muscle intrinsic regenerative capacity, provoking the replacement of muscle fibres by fibrotic and adipose tissue, resulting in muscle weakness and dysfunction [9]. Although this is the most widely accepted hypothesis describing the molecular events in muscular dystrophies, additional explanations such as increased sarcolemma permeability [10], the loss of control over the mechanisms that regulate SC activation [11], defective vasodilatation [12] or general loss in Ca^2+^ homeostasis [5] cannot be ruled out (Figure S1).

The constant development of new pharmacological, cell and gene-based strategies [1] to treat and cure Duchenne patients urges the establishment of reliable methods and standardised protocols to objectively evaluate the benefits of these experimental approaches [13]. While in vivo behavioural tests are an essential source of information to assess general motor functioning [14,15,16,17], in vitro histopathological examination provides the researcher a much more accurate tool to quantify the expression levels and subcellular localisation of diverse proteins related to the disease progression in individual muscle groups [18]. Classical histochemical dyes such as haemotoxylin and eosin (H&E), Picrosirius red (PR), Masson’s trichrome (M’sT) or oil red O (ORO) have been extensively used to assess fibre size distribution, nuclear location, immune cell infiltration, fibrosis or fat accumulation. Nevertheless, immunohistochemistry (IHC), and, in particular, immunofluorescence (IF), has permitted the spatial localisation of multiple degenerative or regenerative markers simultaneously, thus contributing to the comprehension of the cellular and molecular events leading to fibre deterioration and death in Duchenne models [19]. Having such a myriad of possible markers, each of which providing different purposes, with distinct rationale and specific staining protocols, could be puzzling for the researcher. Hence, the aim of this review is to summarise and explain the principles behind some of the most valuable histological techniques available to investigators to quantitatively evaluate the degree of degeneration or regeneration on dystrophic muscles either from patients’ biopsies or samples from animal models. Throughout the article, the reader will find references to articles describing the methods applied for each of these techniques, therefore, the step-by-step protocols are out of the scope of this review.

## 2. Animal Models

As human muscle biopsy is invasive and difficult to reiterate during the disease evolution, there is a need for animal models to better understand the disease physiopathology and to test possible therapies. Thus, throughout the years different models were developed, each of them having their own advantages and drawbacks.

In terms of mammalian models, *mdx* mice are probably the most frequently used [20]. This mouse line (C57BL/10ScSn) has a spontaneous nonsense point mutation (C to T transition) in exon 23 of the *Dmd* gene, which leads to the absence of the full-length dystrophin [21]. However, their phenotype is different from humans, as they only display moderate muscle weakness of the extremities. This difference could be explained by an apparent sustained capacity for muscle regeneration partially due to the increased expression of another extracellular matrix (ECM) anchoring protein named utrophin, which is similar in structure and function to dystrophin. This compensating strategy is only present in mice, as utrophin expression in humans remains insufficient [22]. Similarly, the enhanced expression of the laminin receptor α7β1 integrin observed specifically in *mdx* mice, and to a lesser extent in patients with DMD [23], may partially compensate for the absence of the dystrophin glycoprotein complex in the animal model [24]. Moreover, young *mdx* mice show an adaptation of the bioenergetic apparatus that masks the course of the disease. This is manifested in an increase in the efficiency of Ca^2+^ accumulation and transport in mitochondria [25,26,27], overactivation of respiration and the functioning of the OXPHOS system [28]. As a result, in *mdx* mice, severe dystrophic phenotypes, such as muscle wasting, scoliosis and heart failure, do not appear until the age of 15 months as their lifespan is only reduced by 25% [29]. To worsen the phenotype, double knockout dystrophin/utrophin *(mdx/Utrn^−/−^*) [30] or α7-integrin/dystrophin [23] mice were developed. Both models are more severe than *mdx* mice [30], as the double knockout mice usually die from respiratory insufficiency at the age of 20 weeks [31]. Alternatively, to study exon 2 duplication, accounting for approximately 11% of DMD causative mutations, the *dup2* mice were specifically developed [32]. This mouse model is similar to *mdx* in terms of disease time evolution, pathological findings and phenotype severity and could be used to study duplicated exon-skipping therapies [32].

Among the non-rodent mammals, the most studied model is the golden retriever muscular dystrophy dog. This model reproduces the human disease better than the *mdx* mice, as cardiomyopathy and respiratory failure are the main causes of death, usually at the age of 3 years old, indicating a 75% reduction of normal life expectancy (reviewed by [33]). However, dog models have important disadvantages such as the cost, breeding difficulties and the small number of specimens available, limiting statistically reliable results.

Currently, the perfect animal model does not exist, so the search for optimal and more rigorous models for research continues. In this sense, the use of genome editing methods, such as CRISPR-Cas9, will certainly lead to new animal models becoming increasingly close to humans [34]. For a review on DMD animal models, see [35].

## 3. Histological Methods to Assess Degeneration/Regeneration in Dystrophic Muscles

**Figure 1:**
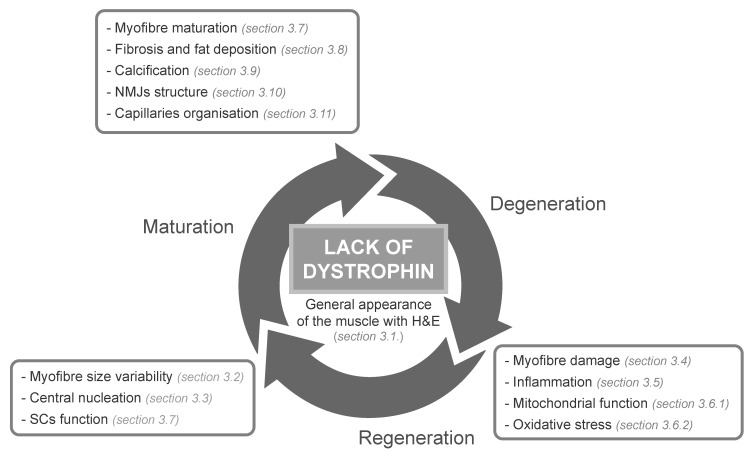
Histopathological examination in dystrophic muscles. Main events and processes that can be assessed through histological tests in muscles lacking dystrophin. These muscles go through multiple rounds of degeneration/regeneration/maturation, ultimately leading to impaired regeneration, chronic inflammation and replacement of muscle fibres with connective tissue, thus producing fibrosis, fat deposition and muscle wasting. Each of these events will be explained in the sections indicated in the figure.

Histopathological examination of dystrophic muscles should start with an evaluation of the general appearance of the muscle with a simple staining such as H&E (Section 3.1, Figure 1), from which a lot of information can be inferred. This staining and other more sophisticated IF markers can be useful tools to study myofibre size distribution (Section 3.2, Figure 1) and the percentage of centrally nucleated fibres (CNFs) (Section 3.3, Figure 1), both parameters providing information about the regenerative process in that muscle. Thereafter, more specific histological techniques can be used to quantify myofibre damage (Section 3.4, Figure 1) and to study the inflammatory process (Section 3.5, Figure 1) through quantification of immune cell infiltration and the presence of inflammatory cytokines (Section 3.5.1 and Section 3.5.2, respectively). In dystrophic muscles, sarcolemmal rupture and increased permeability produce loss of Ca^2+^ homeostasis, mitochondrial dysfunction and oxidative stress. These damaging events can also be evaluated following histochemical and immunohistological (IH) protocols (Section 3.6, Figure 1). In parallel, some cytokines released by immune cells contribute to the activation of SCs, yielding new myofibres, and fibro/adipogenic progenitors (FAPs), which upon differentiation reconstruct the ECM. The progress of SC activation, proliferation, differentiation and myofibre maturation can be followed due to the expression of different markers characteristic of each of these phases (Section 3.7, Figure 1). In dystrophic muscles, after several cycles of degeneration/regeneration/maturation, the initial inflammatory response becomes chronic, FAP proliferation and differentiation are not well regulated, regeneration fails and muscle tissue is substituted by fibrotic, fat and calcified connective tissue (Section 3.8 and Section 3.9, Figure 1). In contrast, in healthy muscles or in dystrophic muscles under certain promising therapeutic interventions, the regenerative phase is culminated with the myofibres’ full maturation due to neuromuscular junction (NMJ) re-establishment and capillaries’ functional reorganisation (Section 3.10 and Section 3.11, respectively, Figure 1). Each of these later events can be studied with different histological tests explained in detail below.

It is worth mentioning that for some muscle histological tests, such as morphological studies, the use of fresh frozen sections is strongly recommended [36]. Whole-muscle fixation with formalin or paraformaldehyde and paraffin embedding, although required for certain histopathological analyses [37,38], needs handling of toxic chemicals, and could be the source of staining artefacts, fibre length deterioration and physicochemical modifications that lead to masking of some tissue antigens [39,40]. On the other hand, preparing and freezing muscle samples require knowledge and practice, as when the freezing procedure is not carried out correctly, the presence of ice crystals inside the myofibres will yield suboptimal muscle cryosections for histological studies. For detailed protocols, the reader is referred to the following articles: [36,39,41].

### 3.1. Evaluation of the General Appearance of the Muscle with Haematoxylin and Eosin

**Figure 2:**
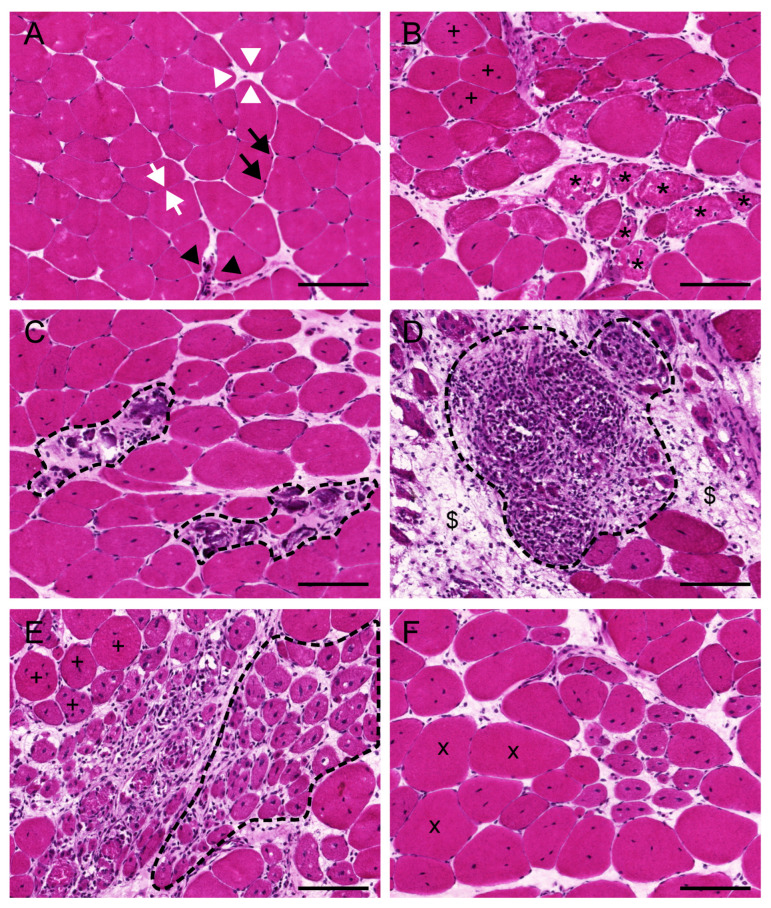
Pathological changes seen in the rectus femoris muscle of *mdx* mice stained with haemotoxylin and eosin. (**A**) General appearance of muscle fibres in a 3-month-old wild type (WT) animal. Fibres (pink) show similar sizes and little connective tissue within the perimysium (white arrows) and the endomysium (white arrowheads). Nuclei (dark purple) are located at the periphery of the muscle fibre (black arrows) and capillaries are evident (light pink) within the endomysium (black arrowheads). (**B**) Clusters of necrotic fibres with basophilic inflammatory cells inside (dark purple) and fragmented sarcoplasm (*) surrounded by regular fibres and regenerative fibres (+) in an age-matched *mdx* mouse. (**C**) Mild and (**D**) severe inflammatory cell infiltration, respectively, phagocytising necrotic fibres (black dashed line), surrounded by fibrotic tissue ($). (**E**) Clusters of small rounded centrally nucleated fibres in similar, early, stage of regeneration or immaturity (black dashed line) adjacent to a group of medium-sized regenerating fibres in later stages of maturation (+). (**F**) In some areas of the muscle, non-centrally nucleated hypertrophic fibres (x), larger than the fibres seen in WT muscles, can be observed. Scales: 100 μm.

H&E are the gold standard stain for muscle samples due to the large amount of information the researcher can obtain with this routinely used dye. For a full description of the H&E protocol and later image acquisition, the reader is referred to [39] or [42]. H&E reveal the overall appearance of the tissue in which the fibres (pink), their contour, size, location of the nuclei (purple) and, in some cases, their condition (necrotic vs. healthy) can also be distinguished. This dyeing method also highlights the presence of inflammatory cells, blood vessels, nerves bundles, muscle spindles and connective and adipose tissue (Figure 2).

Fibre size variation is one of the most prominent features of dystrophic muscles [43]. While in healthy muscles, fibres have roughly similar size, peripherally located nuclei and almost no connective tissue in the perimysium and endomysium (Figure 2A), muscles from patients with DMD and animal models of the disease show highly variable diameters of their myofibres. Depending on the age and degree of physical activity of the individual, some or all of the following features are evident in these muscles: clusters of necrotic fibres with basophilic inflammatory cells inside and fragmented sarcoplasm (Figure 2B), densely stained hypercontracted fibres (Figure 2B), mild or severe cell infiltration (Figure 2C,D, respectively), groups of small rounded muscle fibres in an early stage of regeneration or immaturity (Figure 2E), normally scattered with medium-sized healthy fibres and hypertrophic ones (Figure 2F) [19]. Since early regenerating fibres or myotubes are formed by the fusion of several activated myoblasts, fibres that have undergone necrosis and regeneration, but are still not fully mature, are centrally nucleated (see Section 3.3) [44]. The presence of fibrotic and adipose tissue within fibres is also evident with this stain (Figure 2D), especially in aged individuals. However, for the evaluation of the latter, more specific dyes must be used (see Section 3.8).

With image analysis software, H&E images can be used to evaluate and quantify the area of the muscle cross section covered by critical histological features such as cell infiltration, fibrosis, necrosis or regenerating fibres [42,45]. However, the correct identification of some of these characteristics in H&E images is tedious and requires practice. Therefore, specific IHC or IF stainings (see below) are recommended to accurately evaluate each of these features.

### 3.2. Evaluation of the Morphometric Features of the Myofibre

**Figure 3:**
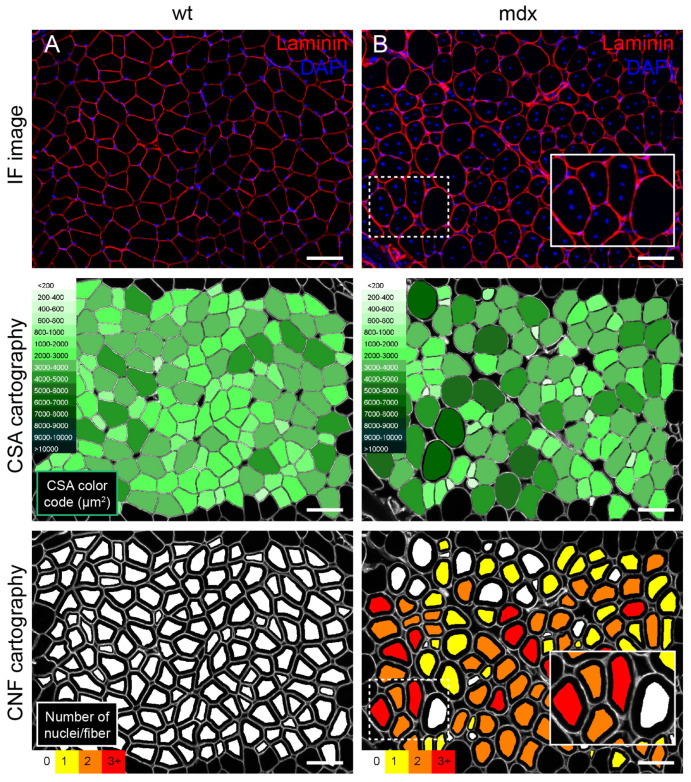
Morphological analysis of the muscle fibres of a wild type vs. an *mdx* mouse. (**A**) Representative image of the quadriceps of a wild type (WT) C57BL/10SCSNJ mouse. The periphery of the myofibres was labelled using an antibody against laminin (red) and nuclei were stained with DAPI (blue) as in [63]. (**B**) Representative image of the same muscle of a C57BL/10SCSN-Dmdmdx J mouse with the same staining. Below both images, the corresponding fibre cross sectional area (CSA) cartography automatically obtained after the analysis of the images shown in A and B with the program MuscleJ ([55], see Table S1). The green-scale colour code indicates the myofibre CSA in μm^2^. This program also quantifies centrally nucleated fibres (CNFs) and the number of nuclei per fibre. At the bottom of the figure, the automatically obtained cartography shows a colour code depending on the number of central nuclei quantified in each fibre (white for 0, yellow for 1, orange for 2 and red for 3 or more nuclei). Note that *mdx* muscle samples are characterised by an irregular distribution of fibre CSA and high numbers of CNFs. Scales: 100 μm.

The analysis of myofibre size distribution across the entire transversal area of the selected muscle is a critical parameter to assess muscle health and regenerative capacity. While in healthy muscles, the majority of fibres within one muscle present roughly the same size, in dystrophic muscles, having newly regenerated fibres intermingled with hypertrophic fibres that have never degenerated produces a great variability in myofibre size [43]. Quantifying the cross sectional area (CSA) of several hundreds of myofibres within a muscle is the standardised method to assess size distribution. Nevertheless, to overcome possible errors in area estimation when muscles are not perfectly sectioned perpendicular to the fibres’ length, calculating the minimal Feret’s diameter (MF’sD) of these same myofibres is strongly advised [46,47]. These parameters, CSA and MF’sD, can be manually estimated tracing the boundaries of fibres in H&E-stained images with programs such as ImageJ [48]. However, this method is arduous and time-consuming. Furthermore, it is less precise than machine learning-based automatic detection because the number of fibres sampled is usually lower and, generally, only a few images, not always representative of the whole muscle, are quantified. Moreover, manual estimation introduces the possibility of user-to-user variability, reducing the replicability and reliability of the study. Hence, in the last 20 years, dozens of automated or semi-automated machine learning-based programs have been developed for the accurate and efficient study of the myofibres’ morphometric parameters (see Table S1). Although some programs have been designed to analyse H&E-stained images [49,50], the great majority of them are intended for the analysis of fluorescence-based images with markers that outline the contour of individual myofibres. This method permits an intensity-based, automatic and unbiased segmentation of hundreds of fibres simultaneously. Commonly, membrane border is identified by IF staining with antibodies detecting basal lamina or sarcolemmal membrane proteins (Figure 3 and Table 1). Undoubtedly, the gold standard marker in skeletal muscle studies is the basal lamina protein laminin [51], however, other proteins have also been used in the literature (see Table 1).

In the context of DMD, as dystrophin is the mutation-affected protein, immunohistology against this marker should only be intended to assess the presence of sporadic or therapy-induced dystrophin-positive revertant fibres (RFs, Section 3.12) [52]. Moreover, as the mutation on the dystrophin gene also alters the expression of the proteins of DAPC and produces its destabilisation [53], immunodetection of dystroglycans, sarcoglycans, decorin or biglycan as markers of the myofibres’ boundaries should be avoided when assessing morphometric parameters of dystrophic myofibres. Moreover, since collagen expression also changes as the disease progresses, this marker is not suitable either. Non-IH procedures are also possible, such as the use of fluorescence-tagged wheat germ agglutinin (WGA), a lectin that binds plasma membrane glycoproteins, thus delimiting the myofibre sarcolemma [54]. For complete protocols on the use of periphery markers for skeletal myofibres, see Table 1.

**Table 1:** Muscle fibre peripheral markers.

Marker	Myofibre/ ECM Location	Technique	Subunits or Types	Morphometry Measurements on DMD	Reference for Full Protocol
Laminin-211	BL	IHC, IF	α, β, γ	+	[55]
Spectrin	SM	IHC, IF	-	+	[52]
Perlecan	BL	IHC, IF	-	+	[56,57]
Dystroglycans	SM/BL	IHC, IF	α, β	-	[58]
Sarcoglycans	SM	IHC, IF	α, β, δ, γ	-	[58]
Dystrophin	SM	IHC, IF	-	-	[52,58]
Collagens	ECM	IHC, IF	I, IV, VI	-	[59,60]
Decorin	ECM	IF	-	-	[60]
Biglycan	ECM	IF	-	-	[60]
WGA	SM	HC	-	+	[54,61]

Different proteins and lectins used to mark the periphery of myofibres for morphometric studies. Some of these molecules are located in the sarcolemma and others are components of the extracellular matrix. Note that not all these markers are suitable for studies on dystrophic muscles, as the expression or location of the proteins may be altered when the dystrophin is absent. Abbreviations: SM: Sarcolemmal membrane. BL: Basal lamina. ECM: Extracellular matrix. IHC: Immunohistochemistry, HC: Histochemistry, IF: Immunofluorescence.

### 3.3. Evaluation of Centrally Nucleated Fibres

As mentioned above, another important parameter to assess the degree of regeneration in a muscle is the analysis of the percentage of CNFs. In healthy adult muscle, nuclei are positioned maximising the distance to each other at the periphery of the myofibre, permitting the transcriptional and translational nuclear products to be uniformly distributed all along the myofibre length. Moreover, this location could protect the nuclei from the contracting forces exerted by the sarcomeres and could prevent these organelles from acting as physical obstacles impeding muscle contraction and function (reviewed by [62]). Several myopathies are characterised by mispositioned nuclei and among them are dystrophinopathies.

Noteworthily, although most of the programs described in Table S1 are freely available, they are all highly variable in terms of their requirements, possibility of visual inspection and later correction, output results and, finally, but not less important, user-friendly interface, ease of use and existence of written or video tutorials that facilitate the comprehension of the analysis process. Therefore, Table S1 intends to help the researcher determine which program to use depending on the needs and previous computational skills.

The most accepted hypothesis explaining the existence of CNFs states that these are fibres going through the regenerative process. During development, multinucleated skeletal muscle fibres are the result of the fusion of mononucleated single myoblasts that only after a maturation phase spatially distribute and extrude the nuclei to the normal sarcolemmal position [64]. In adults, the regenerative process takes place through a similar mechanism, and activated SCs fuse to a pre-existent myofibre that will also become centrally nucleated [44,65]. The movement of these nuclei to the periphery of the regenerated fibre is a complex process that will depend upon the correct functioning and interaction of microtubules and associated proteins, the cytoskeleton, the nucleoskeleton and the linker of the nucleoskeleton and cytoskeleton complex [62].

In the field of DMD, assessing the percentage of CNFs within a muscle has become a standard protocol to quantify and compare the regenerative potential of a novel drug or genetic therapies in dystrophic animal models [47]. As mentioned before, this can be easily achieved in fluorescent cross section samples co-stained with a nucleus-specific dye, such as 4′,6-diamidino-2-phenylindole (DAPI) or Hoechst 33342, and a marker of the periphery of the myofibre (Figure 3). Most of the automated and semi-automated programs explained in Table S1 are also designed to easily quantify the number of CNFs. Alternatively, this parameter can also be assessed manually in H&E-stained muscle cross sections, although the task is labour-intensive.

The percentage of CNFs after an intervention should be carefully interpreted and in parallel with other markers of regeneration/degeneration [66], as lower percentages of CNFs could also be understood as a good prognosis of the disease [67,68]. Moreover, translational studies must acknowledge that this parameter is different in patients with DMD when compared with animal models of the disease [31] and it evolves differently in distinct muscles [45] and with age [69].

### 3.4. Myofibre Damage and Cell Death

In contrast to other myopathies, apoptosis does not seem to have a relevant contribution to muscle wasting in DMD [70]. In patients with DMD, according to the sarcolemma or “microtear” hypothesis [6] (Figure S1), muscle damage is induced by mechanical stress on the already fragilised cell membrane of myofibres due to the lack of dystrophin. This impairment leads to the continuous leakage of molecules such as damage-associated molecular patterns (DAMPs) from damaged myofibres and prolongs the activation of the innate immune response, resulting in chronic inflammation. DAMPs are key factors regulating the priming step that will thereafter activate the NLRP3 inflammasome pathway, will induce the release of proinflammatory cytokines and, ultimately, will cause a highly inflammatory form of programmed cell death called pyroptosis [71]. In addition to pyroptosis, other cell death mechanisms could also contribute to overall muscle wasting in DMD. In this regard, the study by Morgan et al. [7] demonstrated that the molecular pathway responsible for a type of inflammation-induced necrosis, named necroptosis, is activated upon sustained elevation of intracellular Ca^2+^ concentration, supporting the hypothesis of “Ca^2+^ overload” (Figure S1) as another major factor contributing to Duchenne physiopathology [72].

Several histological methods are available to precisely quantify muscle fibre membrane rupture and subsequent cell death. Among them, H&E staining (Figure 2) and Evan’s blue dye (EBD) were the most frequently used techniques in the past [73,74]. Evan’s blue is a membrane-impermeant salt that has been used to assess loss of sarcolemmal integrity due to plasma membrane disruptions [74]. The rationale behind EBD labelling damaged myofibres was based on its ability to form tight complexes with the serum protein albumin, a protein that is taken up by muscle fibres when their sarcolemma is damaged [75]. More recently, however, demonstration that cell staining with EBD results from dye influx via hemichannels formed by connexins [76] and a Ca^2+^-induced increase in membrane permeability [10], and not only from sarcolemmal tears as interpreted before, has triggered the use of different methods such as the staining of serum proteins with molecular sizes above the exclusion limit of hemichannels.

**Figure 4:**
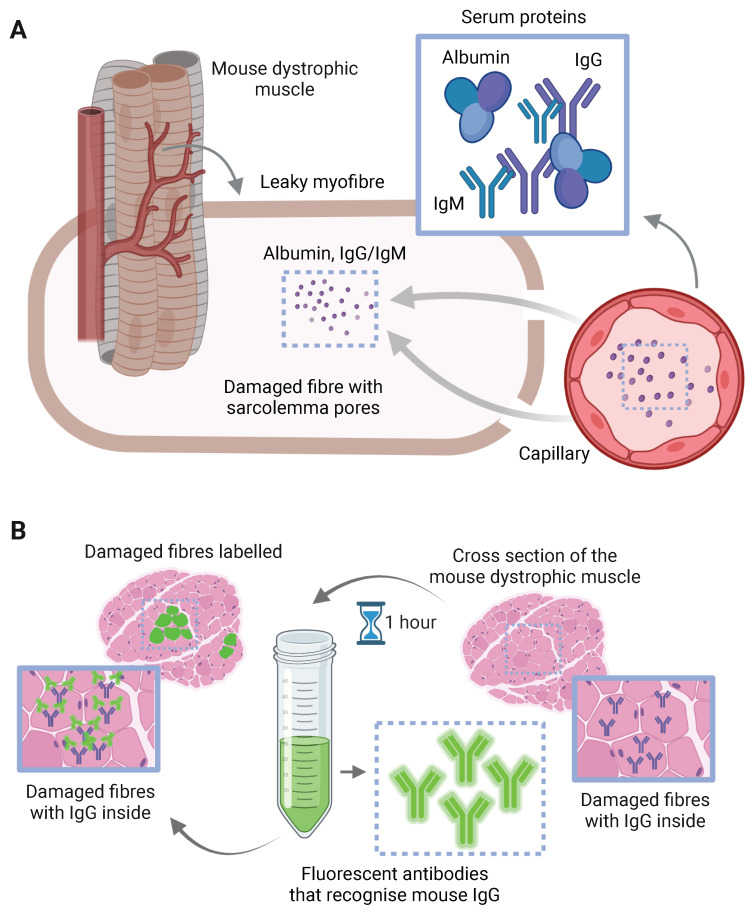
Molecular mechanism explaining the staining of degenerating fibres with IF of IgG. (**A**) Dystrophic muscles have damaged myofibres with sarcolemmal ruptures or pores due to the lack of functional dystrophin. The size of these pores is big enough for serum proteins such as albumin or immunoglobulins (Igs) to enter the leaky membrane and stay in the cytoplasm. (**B**) To recognise these degenerating fibres, incubation of dystrophic mouse muscle cross sections with a solution containing a fluorescently tagged antibody recognising mouse IgG will yield labelling of the myofibres that contain those Igs in their cytoplasm.

Thus, only cells having large sarcolemmal disruptions will show deposition in their cytoplasm of high molecular weight serum proteins such as albumin or immunoglobulins (Ig) (Figure 4). Hence, a simple IHC or IF of albumin could reveal the presence of those damaged fibres [77]. Similarly, the myofibres with membrane disruptions can be easily recognised using an antibody to the same species IgG/IgM tagged with a fluorescent molecule [78] (Figure 4).

Interestingly, some of the programs described in Table S1 can quantify these Ig-positive fibres with the same method used to evaluate fibre type proportions (see Section 3.7). The expression of these results varies from group to group, but most frequently they are expressed as the area of the muscle section occupied by immunopositive damaged fibres [7] or the percentage of Ig-positive fibres [79].

### 3.5. Inflammation

As mentioned earlier, the continuous release of cytoplasmic contents from the dystrophic muscle cells produces the activation of the innate immune system. Once released into the extracellular space, DAMPs bind specific receptors that in turn influence the recruitment and function of immune cells at the site of damage in dystrophic muscle [80]. In healthy muscle, local activation of the innate immune response is essential for necrotic fibre clearance, SC activation, ECM remodelling and complete regeneration [81]. However, in the dystrophic muscle, the continuous focal bouts of myofibre degeneration prolong the inflammatory process (Figure S1) and this chronic state ultimately leads to myofibre death, fibrosis, fat deposition and muscle wasting [2,3].

#### 3.5.1. Immune System Cell Infiltration

Upon damage, muscle regeneration depends on the orchestrated infiltration of different immune cells among which neutrophils, macrophages and T cells play a crucial role in DMD pathogenesis [80]. When sorting and identifying immune cells, the researcher must acknowledge that cell-identifying specific antigens may vary between species, thus appropriate antibodies must be chosen depending on the cell type to be identified and the source of the muscle tissue.

- Neutrophils:

Neutrophils are one of the first granulocytic myeloid cells recruited after muscle injury. In healthy individuals, neutrophils clear up cell debris and are the first line of defence against invading microorganisms. Moreover, they aid in the recruitment of other immune cells such as macrophages. In DMD, DAMPs released from damaged myofibres promote degranulation of mature neutrophils, releasing several antimicrobial enzymes from azurophilic granules. These granules contain neutrophil elastase, that induces chromatin decondensation, myeloperoxidase (MPO), that catalyses the production of reactive oxygen species (ROS) including hypochlorous acid, and neutrophil extracellular traps, that are released outside the cell and further promote inflammation [2,3]. Therefore, neutrophils contribute to prolonging inflammation in Duchenne pathology by releasing proinflammatory cytokines and other compounds that lead to oxidative stress.

Although Ly-6G, an anchored surface protein implicated in tissue neutrophil extravasation, is the most widely used marker to recognise mouse neutrophils by immunohistology [82], both mouse and human neutrophils have also been identified in the literature with antibodies against Ly-6B2 or enzymes contained in their granules, such as MPO (Table 2), albeit never in dystrophic muscles.

- Macrophages:

Macrophages are probably one of the most important players in innate immunity. In muscle, they have several roles such as defence against potentially damaging molecules, tissue repair and regeneration [83]. By oversimplifying, macrophages could be divided into two different subtypes: the proinflammatory M1 macrophages involved in the responses against pathogens and tissue injury and the anti-inflammatory (M2) types being involved in wound healing and tissue repair. This binomial denomination represents the two ends of a wide spectrum [84]. This heterogeneity of macrophages poses a serious challenge for quantitative and qualitative analyses in muscle tissue. In addition, there is a wide variety of macrophage markers for IHC and IF (see Table 2); the choice of which will depend on the subset of macrophage, and the tissue targeted. Moreover, there are very few unique macrophage markers and normally a co-staining will be required to identify the specific population of macrophage required.

If the objective is to determine the absolute number of macrophages, a pan-macrophage marker is preferred. CD11b, CD11c, F4/80 and CD68 are commonly used in IHC and/or IF [85]. Both CD11b and CD68 staining have been shown to be equally efficient as pan-macrophage markers in human skeletal muscles [86] and can also be used in mouse muscles (Table 2). However, they are also expressed in other cell populations, thereby limiting their specificity and overestimating the macrophage population [87].

F4/80, to the contrary, is a glycoprotein which specifically marks murine macrophages [88]. Interestingly, the antibodies to F4/80 do not recognise fibroblasts, polymorphonuclear cells or lymphocytes, which usually share the same receptors and surface antigens as macrophages [89]. However, it does mark Langerhans cells [90] and eosinophils [91], making it less specific. Nevertheless, the utility of F4/80 as a marker for tissue macrophages has been confirmed in various organs, including mouse muscles (Table 2).

The advance in terms of IH techniques has allowed scientists to identify specific macrophage subpopulations. Besides the functional definition of M1 and M2, cell surface markers have been identified to distinguish these two populations. In IF, co-staining of a pan-macrophage marker with inducible nitric oxide synthase (iNOS) has shown good results for identifying M1 macrophages, while CCR2, CCR7, CD80 or COX-2 are also good M1 markers that may be used alone (Table 2). M2 macrophages (Table 2), on the other hand, have been shown to express high levels of CD163, CD206 and Arg-1 [92]. CD206, the mannose receptor, is a well-accepted M2 macrophage marker in skeletal muscle [92], although its staining might also be present on other cell types such as SCs [93].

Finally, it is important to keep in mind that results from studies of skeletal muscle macrophages in animal models are informative. The macrophage markers that are used in mice either do not exist or identify different populations in humans, complicating the extrapolation of findings from mouse models to human studies [94].

- Lymphocytes:

The inflammatory infiltrates found in dystrophic muscle are heterogeneous. Besides neutrophils and macrophages, T cells are also present. Indeed, CD4+ (helper) T cells and CD8+ (cytotoxic) T cells increase in the blood of patients with DMD [95] (Table 2). A recent theory even suspects that interferon gamma (IFNγ) in dystrophic muscles is mainly produced by CD4+ effector T cells and not by macrophages, thereby highlighting the main contribution of lymphocytes in DMD pathophysiology [96].

Another type of T cell are the regulatory T cells (Tregs) which maintain immune homeostasis [97] via the secretion regulatory cytokines such as interleukin 10 (IL-10), being thereby able to control T effector, B and antigen presenting cell (APC) activation and proliferation [98]. While T cells are usually detected by the CD4, CD8 and CD3 markers (see Table 2), Tregs can be identified by the co-expression of CD4, with CD25, or Forkhead box protein 3 (FOXP3) (Table 2).

#### 3.5.2. Cytokines

Although cytokine levels are normally measured by RT-qPCR or Western blotting (WB) in tissue homogenates, histological location of the source of those cytokines and quantification of their expression in muscle cross sections are also possible. Currently, a wide range of antibodies targeting inflammatory cytokines are available on the market either for IF or IHC (Table 2).

- Proinflammatory Cytokines:

In DMD, degenerating dystrophin-absent fibres secrete proinflammatory cytokines and chemokines, implying the continuous recruitment of the above-mentioned immune cells which in turn activate M1 macrophages, leading to a chronic inflammatory state producing high concentrations of proinflammatory cytokines such as IFNγ, tumour necrosis factor alpha (TNFα), interleukin 1 beta (IL-1β), interleukin 18 (IL-18) and interleukin 6 (IL-6) (Table 2) [98,99]. IL-1β and TNFα are key proinflammatory cytokines involved in initiation and perpetuation of muscle pathology in DMD. In mdx mice, IL-1β mRNA levels have been shown to be higher than in controls [100], while TNFα significantly increased with age in the diaphragm muscle [101] and in sera of most patients with DMD [102]. IL-6, on the other hand, is a pleiotropic cytokine having several roles encompassing immune response, haematopoiesis, and inflammation regulation [103]. This cytokine increases in patients with DMD compared with healthy subjects [104]. Interestingly, inducing IL-6 expression in *mdx* mice exacerbates the dystrophic muscle phenotype, resembling the severity observed in humans [105]. Thus, IL-6 blockade exerts a positive effect on muscle regeneration in dystrophic mice [106]. This same group observed with IF against IL-6 an increase in its expression in muscle biopsies from patients with DMD when compared with healthy subjects, a result that was later confirmed by RT-qPCR [105].

**Table 2:** Markers of the inflammatory process in rodent muscles.

Cell/Cytokine	Type	Antigen/Marker	IH Method	Tissue	Animal	References for Protocol
**Macrophages**	Pan-marker	CD11b	IHC/IF	Skeletal muscle	*mdx* mice	[107,108]
		CD11c	IHC			[107]
		CD68	IHC/IF			[109]
		F4/80	IHC/IF			[110]
	M1	CCR2	IHC/IF			[109,111]
		CCR7	IHC		*CCL19KL* mice	[112]
			IF		Rat	[113]
		CD80	IHC		Rat	[114]
		iNOS	IHC/IF		*mdx* mice	[110]
		COX-2	IHC			[115]
	M2	Arginase-1	IF			[116]
		CD206	IHC/IF			[109]
		CD163	IHC/IF			[109,117]
**T cells**	Pan-marker	CD3	IHC/IF	Skeletal muscle	*mdx* mice	[118,119]
	T cell helper	CD4	IHC/IF			[109,120]
	T cell cytotoxic	CD8	IHC/IF			[107,120]
	Treg	FOXP3	IHC/IF			[121]
		CD25	IF			[120]
**Neutrophils**	/	Ly6B2	IHC	Skeletal muscle	*mdx* mice	[109]
		Ly6G	IHC/IF			[110,119]
		MPO	IF		*SOD* mice	[122]
**Cytokines**	Proinflammatory	TNFα	IHC/IF	Skeletal muscle	*mdx* mice	[118,123]
		IL-6	IF			[106]
		IFNγ	IHC			[110]
		IL-18	IHC			[124]
		IL-1β	IHC/IF			[124,125]
	Anti-inflammatory	IL-10	IF	Peripheral nerve		[126]
		IL-4	IHC	Skeletal muscle		[110]

Different antigens and markers used to identify immune cells and cytokines released upon inflammation in skeletal muscles of rodents. Abbreviations appearing in this table are explained in the Abbreviations section.

- Anti-inflammatory cytokines:

IL-10 is an anti-inflammatory cytokine regulating the production of proinflammatory cytokines and the activation of M1 macrophages to reduce further cytokine production [127]. More specifically, IL-10 inhibits the expression of two important proinflammatory cytokines, namely IFNγ and TNFα. In addition, IL-10 specifically inhibits the expression of iNOS by M1 macrophages [117] and activates M2 macrophages [128], which are predominantly represented in *mdx* muscle areas during the regenerative stage of the disease, suggesting its involvement in muscle regeneration [110,117]. While the expression of IL-10 in skeletal muscle has been extensively studied by WB or RT-qPCR [117], to our knowledge, no histological techniques have been used to identify it in muscle tissue. However, IF methods to identify IL-10 exist, as they are used in the peripheral nerve by Mietto et al. [126] (Table 2).

Other anti-inflammatory cytokines such as IL-4 or transforming growth factor-beta (TGF-β) are commented on in Section 3.8.

### 3.6. Mitochondrial and Sarcoplasmic Reticulum Function and Oxidative Stress

#### 3.6.1. Mitochondrial Function

Being a highly metabolically active tissue, skeletal muscle is rich in mitochondria. These organelles are not only responsible for the synthesis of ATP by oxidative phosphorylation but are also essential for Ca^2+^ homeostasis and Ca^2+^-dependent signalling. In DMD, ruptures in the sarcolemmal membrane, increased permeability and, more importantly, deficiencies in the machinery regulating Ca^2+^ stores and Ca^2+^-dependent signals produced by the dystrophin deficiency [5] induce Ca^2+^ overload, mitochondrial swelling [10] and ROS production, leading to mitochondrion-dependent cell necrosis [129,130]. Hence, Ca^2+^ overload and malfunction of muscle mitochondria [131,132] also contribute to chronic inflammation and progressive cell death in DMD physiopathology (Figure S1). Notably, therapeutic interventions directly or indirectly improving mitochondrial function led to reduced inflammation and improved respiration, among other positive physiological effects, but most importantly, they ameliorated in vivo muscle function [133,134,135,136].

Complementary to other recommended techniques such as mitochondrial enzymatic activity assay, bioenergetic assessments, WB, RT-qPCR or mitochondrial respirometry [137], assessing the quantity, appearance and functionality of mitochondria with histological procedures is also possible. For quantification and assessment of morphological abnormalities, transmission electron microscopy (TEM) is recommended [138,139], as general quantification by IHC or IF with antibodies to mitochondrial proteins such as cytochrome C oxidase (COX) or TOM20, although possible [140,141,142], is less precise due to the size of these organelles.

To study mitochondrial function in frozen muscle sections, observing the activity of two respiratory enzymes such as complex IV or COX and complex II or succinate dehydrogenase (SDH) is a widely used approach in different tissues [143]. Since COX catalytic subunits are codified by mitochondrial DNA (mtDNA), assembly and function of the complex depend on mtDNA integrity. To the contrary, the activity of SDH is independent of impaired mtDNA because it is entirely encoded by nuclear DNA. The accumulation of mutations in mtDNA or nuclear DNA due to mitochondrial impairment, disease or ageing leads to the presence of fibres with low or absent COX and SDH activity, respectively. Thus, analysis of this stain is based on the presence of fibres with low, medium or high density of brown/blue products depending on the COX/SDH enzymatic activity, respectively. Consequently, the results are usually presented as the percentage of fibres corresponding to each group within the studied muscle cross section. Using this and other complementary techniques, Moore et al. [137] was the first to show evidence of mitochondrial complex II and IV impairment occurring prior to muscle fibre damage in 11-week-old *mdx* mice.

Additionally, mitochondrial abnormalities can be studied with a modified version of Gömöri trichrome (GT) stain [144]. This protocol permits the visualisation under the microscope of characteristic ragged red fibres due to the accumulation of abnormal mitochondria below the sarcolemma of the myofibre. Moreover, mitochondrial function has also been assessed with the nicotinamide adenine dinucleotide tetrazolium reductase stain (NADH-TR). This method provides a measure of the muscle respiratory capacity because the tetrazolium colourless salt is used as an electron acceptor reduced by the NADH enzyme to an insoluble purple-coloured product wherever this enzyme is active [19]. Contrary to COX/SDH stainings that are specific for mitochondria, GT and NADH-TR stain results should be interpreted cautiously since both dyes also reveal SR. For complete protocols of the latter dyes on mouse muscle samples, please see [145].

#### 3.6.2. Oxidative Stress

As a result of mitochondrial malfunction, dystrophic muscles show an increase in enzymes and markers related to oxidative stress (Figure S1) that can be evaluated with different histological techniques [3]. In frozen sections, quantification of the number autofluorescent ceroid and lipofuscin granules generated due to chronic oxidative stress [146] can be assessed easily and give some important information concerning oxidative stress status [147,148]. Similarly, the fluorescent staining dihydroethidium (DHE), a superoxide indicator, is also representative of the levels of ROS [148,149].

Moreover, several IH protocols have been used in the literature to assess the level of oxidative stress in skeletal muscle sections. Antibodies to 4-hydroxynonenal (HNE), a lipid peroxidation by-product [150], to peroxiredoxin 3, a mitochondrial hydrogen peroxide scavenger enzyme [151], to 8-hydroxy-2′-deoxyguanosine, an antioxidative enzyme [152], or to nitrotyrosine, which is the result of tyrosine nitration occurring only when tyrosine interacts with increased levels of ROS and nitrogen species [153], are all well-known witnesses of oxidative stress. Furthermore, all these markers increase in *mdx* mice and, interestingly, retinol, tempol or adiponectin act as potent antioxidant agents [118,148,154]. For protocols, see the latter references.

#### 3.6.3. Sarcoplasmic Reticulum

Mislocation and dysfunction of the sarcoplasmic reticulum (SR) and its Ca^2+^-regulating proteins also contribute to the loss of Ca^2+^ homeostasis and play an important role in the progression of the disease in dystrophin-lacking muscles [155,156,157,158]. Surprisingly, although SR function has been studied with multiple other techniques [155,158,159,160], histological tests to study the SR are not routinely performed in dystrophin-deficient muscles. Nevertheless, as mentioned earlier, GT, besides staining mitochondria, may also reveal the SR. In fact, this stain has been used in the literature to show the presence of tubular aggregates. These SR-derived morphological abnormalities [161] are characteristic of several myopathies [162,163]. Additionally, assessing the expression and location of SR-specific proteins such as the SERCA pump or ryanodine receptors has also proven to be a useful method to indirectly study SR function and structural abnormalities. This can be easily performed in muscle cryosections [164] or isolated fibres [165,166] and could be an interesting method to study the SR in dystrophic-deficient muscles [164]. As with other organelles such as mitochondria, confirmation of possible structural and locational abnormalities with TEM or with revolutionising new techniques such as dSTROM super-resolution imaging [167] is also advised.

### 3.7. Myofibre Regeneration and Maturation

Skeletal muscle regeneration is a highly coordinated process with sequential but overlapping steps from the inflammatory reaction and macrophage invasion, later activation, differentiation and fusion of SCs and, finally, maturation of the newly formed myofibres [168]. Once the regeneration of new muscle fibres takes place, it is possible to follow the sequential process of fibre regeneration–maturation characterised by the expression of phase specific markers. In healthy muscles, muscle regeneration is a highly ordered process [168], however, in dystrophic muscles the process is not completely synchronised and there are therefore regional differences in the progression of the degenerative/regenerative process within the same muscle.

#### 3.7.1. Satellite Cells and Early Regeneration

Regeneration of damaged dystrophic myofibres depends upon the activation of otherwise quiescent SCs located in close apposition to the fibre sarcolemma and underneath the basal lamina [65]. SC commitment to myogenesis requires the activation of the transcription factor PAX7 and, later, several myogenic regulatory factors, including MYOD, MYF5, MRF4 and MYOGENIN. Upon activation, satellite stem cells undergo either symmetric or asymmetric cell division, contributing either to the maintenance of the stem cell pool or to the proliferation and differentiation of SCs to form regenerative myoblasts, respectively [169].

Historically, the depletion of the regenerative muscle capabilities with age in dystrophic muscles was attributed to the functional exhaustion of the pool of quiescent SCs (“satellite cell exhaustion hypothesis”) [170]. Recently, however, results demonstrating high expression of the protein dystrophin in SCs and its important role regulating SC behaviour [171] have led to an emerging model suggesting that the lack of dystrophin renders dysfunctional SCs unable to contribute efficiently to the myofibre regeneration [169]. Moreover, in dystrophic muscles the unrestrained continuous activation of SCs is known to be the source of weak and fragile branched fibres especially sensitive to eccentric muscle contractions [172,173] (Figure S1).

Accordingly, promoting the ability of dystrophic SCs to correctly enter the myogenic programme and optimising strategies to ensure functional SC delivery are possible therapies for the treatment of DMD [174]. To achieve this, validation with in vitro tests on cell cultures must be complemented with in vivo muscle histological studies. Therefore, to correctly identify quiescent or recently activated SCs in muscle cross sections, recognition of Pax7^+^/Ki67^-^ or Pax7^+^/Ki67^+^ cells, respectively, below the basal lamina by IHC or IF is the gold standard procedure [175,176]. Similarly, the identification of highly proliferative activated SCs, also called myoblasts, is based on the recognition of MyoD [177]. Finally, differentiated myoblasts or myocytes can be labelled with antibodies recognising myogenin [177]. Importantly, the expression of each of these transcription factors and proteins is not an all or nothing process. There is some degree of overlap between different factors and cell stages (reviewed by [178]). Thus, to better distinguish between quiescent or activated SCs, myoblasts, myocytes and myotubes, simultaneous co-staining with different markers is recommended [176,177]. For a full protocol, see the methodological paper of [179].

Finally, the last step of the early-regenerative process depends upon the degree of damage. Myocytes may either bind to a pre-existing myofibre or to other myocytes to form immature myotubes. At this stage, myogenin promotes the expression of immature forms of myosin heavy chains (MyHCs) [180] in the newly regenerated myofibres.

#### 3.7.2. Early Maturation of Muscle Fibres

There are reportedly multiple isoforms of MyHCs, which are expressed in different phases of the skeletal muscle fibre regeneration/maturation process [181]. Consequently, histological identification of these isoforms can be used to characterise the maturity stage of the muscle. During early muscle maturation, the new regenerated fibre is characterised by the re-expression of neonatal (neo-) and embryonic (emb-)MyHCs encoded by the *MYH3* and *MYH8* genes, respectively. Many studies have shown the re-expression of these proteins in the adult following injury or in neuromuscular disorders such as DMD [182,183]. In the pathological context of DMD, IF and IHC studies showed high levels of neo- and emb-MyHCs in different animal models [184,185,186] as well as in patients with DMD [187]. Thus, these proteins have been reported to be a biomarker of muscle damage and DMD severity [187]. Moreover, since muscle regeneration in dystrophic muscles is muscle- and age-dependent [185], emb-MyHC represents a robust specific marker of the degenerative/regenerative process in the DMD context.

Although most commonly used to identify capillary and connective tissue injury in immune myopathies such as dermatomyositis [188], the alkaline phosphatase (ALP) reaction is also used to identify regenerating myofibres in muscle biopsies. This histochemical stain produces a black reaction product due to the activity of the ALP enzyme on an exogenous substrate that reacts with a diazonium salt precipitating at the site of the enzyme activity. The ALP enzyme is primarily found in cell membranes where active transport processes occur. Thus, while in healthy skeletal muscle this enzyme is only present in the endothelium of arterioles but never in capillaries, myofibres or connective tissue [189], different pathological conditions yield distinctive patterns of staining [190]. Hence, the stain highlights darkly stained myofibres scattered among pale yellow ones, and/or a focal black staining in perimysial connective tissue and/or in endomysial capillary, the latter two typically observed in dermatomyositis [191] or antisynthetase syndrome [190], but not in patients with DMD [189]. Importantly, especially for these patients, stained myofibres have been interpreted in the literature either as regenerating or necrotic myofibres, forcing a more specific staining to distinguish between the two.

Alternatively, utrophin, an autosomal paralogue of dystrophin, is also considered as a regeneration-associated protein. The expression of this protein, normally limited to the neuromuscular and myotendinous junctions in the adult, increases upon regeneration in dystrophic muscles from DMD and BMD patients as well as in animal models [192]. This could be a compensatory mechanism capable of improving the membrane stability of dystrophic myofibres, as it suppresses the functional signs of dystrophinopathy [193,194,195]. Moreover, recently, staining studies demonstrated a positive correlation between the expression of utrophin and emb-MyHC levels in dystrophic muscles of *mdx* and dystrophin/utrophin-deficient double knockout (*dko*) mice, supporting the use of utrophin staining as regenerative marker in dystrophic muscles [185]. However, this correlation is animal model-dependent, as an inverse correlation between these two parameters has been demonstrated in *mdx-Fiona* mice [185], a model in which over-expression of utrophin provides a significant functional rescue of the dystrophic phenotype and corrects a large majority of the *mdx* serological biomarkers [196]. In conclusion, and, as mentioned before, when assessing regeneration in dystrophic muscles, the researcher must consider other complementary indices such as CNFs, fibre size distribution, levels of regeneration-associated genes and secreted factors released during muscle repair responsible for guiding muscle regeneration [197].

#### 3.7.3. Late Maturation of Muscle Fibres

After the transient upregulation of the above-mentioned regeneration markers, their expression decreases as maturation progresses. Among the mature healthy fibre markers, the analysis of dystrophin re-expression in an individual fibre allows an accurate assessment of myofibre maturity [198]. However, in the case of DMD where dystrophin is not expressed, other markers of muscle fibre maturity, such as myozenin 1, a Z-disk-associated protein, are needed [199,200]. This protein expression disappears after injury but gradually reappears during muscle regeneration as demonstrated by IF analysis in the study by Yoshimito and colleagues on *D2-mdx* mice [198].

Although skeletal muscle tissue is composed of thousands of muscle fibres, it is important to note that the metabolic and contractile properties of the muscle depend on their fibre type composition [181]. While type 1 fibres (slow oxidative) are abundant in red muscle, type 2 (fast glycolytic) are typical in white muscles. Fibre types can be distinguished by their MyHC ATPase activity as shown by the histochemical method developed by Engel [201]. However, this method is not able to distinguish between 2X and 2B fibres. Thus, an improved method called metachromic ATP staining was developed [202]. Additionally, two other histochemical methods (both explained in Section 3.6) resulting in similar results can be used: NADH-TR or SDH staining [203,204]. Nevertheless, there appears to be a discrepancy between the classification of muscle fibre types based on their myosin ATPase activity and their metabolic properties [205], thus, attempts to combine the two histochemical techniques have generally failed.

Currently, IF or IHC with specific antibodies to each isoform of MyHC is more commonly used since it permits the simultaneous identification of all muscle fibre types in a single muscle cross section. The generation of specific anti-MyHC antibodies has provided a powerful tool not only to define the fibre types present in skeletal muscles, but also their functional properties, their response to conditions that affect muscle plasticity and their changes in muscle disorders [181]. Indeed, many antibodies that specifically recognise MyHC isoforms are already readily available, facilitating the experimental procedure and yielding accurate results that are easier to analyse. For a complete step-by-step protocol for muscle fibre staining and analysis, consult Bloemberg and Quadrilatero, 2012 [206]. Interestingly, the analysis of the percentage of fibre types in a muscle can be carried out manually [63] or semi-automatically with some of the programs summarised in Table S1.

Surprisingly, only some studies have investigated fibre type changes under the dystrophic phenotype and contradictory results have been reported [6,207,208,209]. Nevertheless, in general, fast-twitch fibres seem to be more susceptible to contraction-mediated damage than slow-twitch fibres [209,210,211]. Although the mechanisms that confer higher resistance to type 1 fibres remain speculative [173,212,213], clear higher resistance of slow-twitch fibres in dystrophic muscles have triggered the search for factors and mechanisms to promote a fast-to-slow switch in muscles as a therapy to ameliorate the effects of DMD [214,215]. To demonstrate the efficiency of these treatments, histological assessment of distribution and relative percentages of fibre types in severely affected muscles, such as the diaphragm or the EDL, is necessary.

### 3.8. Fibrosis and Fat Deposition

#### 3.8.1. Fibrosis

Fibrosis due to unregulated deposition of ECM components in muscles of patients with DMD is one of the multiple secondary effects of this devastating disease [216] (Figure S1). Under physiological conditions, ECM forms up to 10% of the skeletal muscle weight and it plays a principal role in force transmission, maintenance and repair of muscle fibres following injury [217]. ECM is mainly synthetised by specific cells called (myo)fibroblasts mostly derived from mesenchymal progenitors named FAP cells [218].

**Figure 5:**
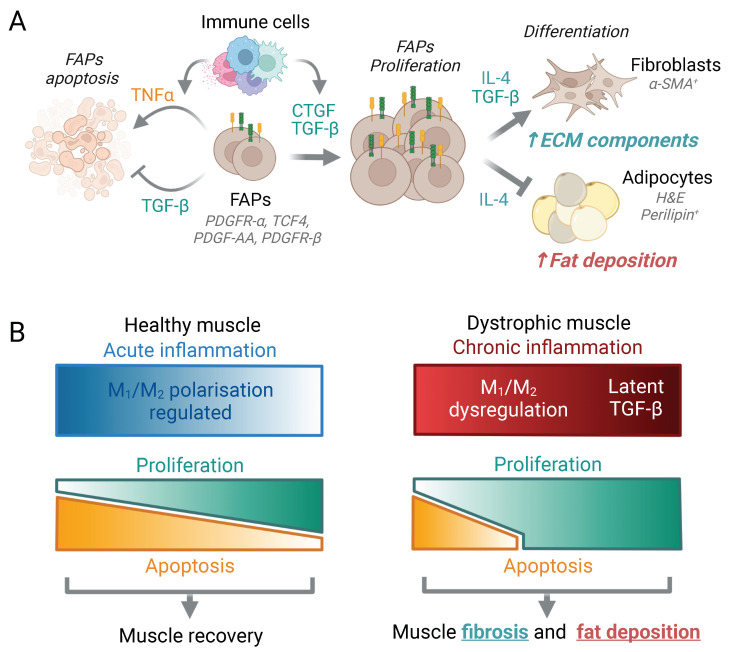
Regulation of connective tissue regeneration in healthy and dystrophic muscles. (**A**) Proliferation and differentiation of FAPs into myofibroblasts or adipocytes (markers in grey cursive letters) are regulated by pro- and antifibrotic signals released mainly by immune cells. While TNFα released by M1 macrophages induces FAP apoptosis [236], anti-inflammatory cytokines such as TGF-β and connective tissue growth factor (CTGF) promote their proliferation and differentiation into ECM-producing fibroblasts [237]. Moreover, eosinophil-produced interleukin 4 (IL-4) also contributes to FAP differentiation, and while activation of the IL-4 signalling cascade promotes mouse FAP differentiation into fibroblasts and contributes to normal connective tissue remodelling, its inhibition produces their differentiation into adipocytes and is associated with increased intramuscular fat, glucose dysregulation and muscle weakening [238,239]. (**B**) In healthy muscle, upon injury, tight regulation of the innate immune response with balanced release of profibrotic and antifibrotic factors produces the correct regeneration and maturation of damaged myofibres and parallel reconstruction of the extracellular matrix (ECM). However, in patients with DMD and animal models of the disease, continuous cycles of degeneration and regeneration contribute to chronic inflammation, constant release of profibrotic TGF-β and dysregulation of the ECM component deposition, leading to substitution of muscle fibres with fibrotic and adipose tissues.

Understanding the mechanism of muscle fibrosis is essential to further develop novel therapeutics against this process [216]. Thus, tight regulation of FAP proliferation and differentiation into myofibroblasts or adipocytes is key to control excessive ECM deposition and fat accumulation, respectively (Figure 5A). In dystrophic muscles, chronic inflammation and dysregulation of M1 to M2 polarisation induce a latent increase in profibrotic signals, leading to dysregulation of FAP proliferation and differentiation, constant ECM component deposition and fibrosis [80] (Figure 5B). Nevertheless, contrary to what happens in patients with DMD, aged *mdx* mouse fibrosis is only developed in the diaphragm and scarcely in limb muscles [219]. Thus, the latter muscles of the *mdx* model are not recommended for evaluation of the effectiveness of fibrosis treatments.

The extent of fibrosis in skeletal muscle is typically quantified using classic dyes such as Picrosirius red (PR) or Masson’s trichrome (M’sT) that define the fractional area of a muscle cross section that is occupied by the ECM [220,221,222]. Both PR and M’sT are technically simple and quick to perform and provide images suitable for automated quantification (see [221] for detailed M’sT analysis explanation). Additionally, IHC or IF techniques can also identify the degree of fibrosis in the tissue by targeting different elements involved in the process such as profibrotic factors (1), FAPs and myofibroblast markers (2) and ECM proteins (3), which are three sequential steps in the development of fibrosis.

First, regarding profibrotic factors, once muscle is injured, different cell types secrete TGF-β isoforms, a multifunctional cytokine involved in a variety of cellular processes, including myofibre repair and the regulation of connective tissue formation (Figure 5). Corroborating the excess fibrosis in dystrophic muscles, studies based on IHC analysis demonstrated that the expression of TGF-β1 is specifically upregulated in the muscle cell sarcoplasm and myenteric interstitium of patients with DMD compared to controls, and its expression correlated with their degree of severity [223,224]. Similar results were obtained by IF in muscle sections of *mdx* mice [225]. Moreover, since the activation of the TGF-β signalling pathway produces fibrosis in skeletal muscles, recognition of the phosphorylated form of SMAD2/3 (p-smad2/3), a downstream TGF-β target, has been studied by IF in muscle biopsies of patients with DMD [226], as well as in skeletal muscle of *mdx* mice [227], demonstrating in both cases its correlation with fibrosis. In addition, the effect of TGF-β may be associated with the expression of its target genes such as connective tissue growth factor (CTGF), also considered as profibrotic factor able to reproduce and amplify TGF-β’s effect on fibrosis [228]. Indeed, like TGF-β, IHC analyses have demonstrated that the level of CTGF is specifically upregulated and significantly correlated with the degree of severity of patients with DMD [224,229] and in *mdx* mice [227].

With respect to the cells producing the ECM components in skeletal muscle, FAPs expressing cell-surface platelet-derived growth factor receptor-α (PDGFR-α) and transcription factor 4 (TCF4) seem to be responsible for connective tissue synthesis and are thought to be the origin of myofibroblasts [230]. Thus, this cell population can be tracked in muscle cryosections by IF with antibodies against PDGFR-α or TCF4 [231]. Using these same markers, it has been shown that skeletal muscle resident mesenchymal progenitors expressing PDGFR-α can differentiate into fibroblasts in vitro [232] and that constitutively active PDGFR-α knockin mice show induced fibrosis in skeletal muscle [233]. However, PDGFR-α is not only an excellent marker of FAPs, but also a key functional molecule in the progression of muscle fibrosis. Indeed, a ligand of PDGFR-α, PDGF-AA, promoted the proliferation of PDGFR-α+ cells to the same degree as TGF-β [218], supporting the use of PDGF-AA as another fibrosis marker in muscle sections [234]. Finally, although less studied, the PDGFR-β isoform and its ligand also represent a marker of fibrosis as demonstrated by IF and IHC analysis in muscle biopsies from patients with DMD [235]. As mentioned before, IF analysis of TCF4 has also been used to evaluate fibrosis in skeletal muscles. While Contreras and colleagues [231] showed a direct correlation between the number of positive cells, total amount of TCF4 and the degree of fibrosis in different mdx muscles, Pessina and colleagues demonstrated higher TCF4 immunoreactivity in muscle biopsies from patients with DMD when compared with healthy patients [226].

Myofibroblasts, on the other hand, have been identified in the literature by their expression of alpha smooth muscle actin (α-SMA). However, the contribution of this subset of myofibroblasts to fibrogenesis varies among different tissues [240]. Although this marker has been generally used to identify fibroblasts in *mdx* mice skeletal muscles, sometimes as a single marker [154], other times in conjunction with a reporter GFP transgene co-expressed in collagen type 1 expressing cells [234], a recent study shows that α-SMA is not a good functional marker of fibrogenic cells in the fibrosis process associated with muscular dystrophy [241]. Moreover, the protein α-SMA is also expressed by smooth muscle cells surrounding blood vessels [12].

Finally, IF analysis targeting proteins expressed in the ECM, such as collagen (type I and type III) and fibronectin, also suggests good indicators of fibrosis and is very often used [220]. Many studies have shown increased expression of fibronectin and collagen type I and type III in fibrotic muscle from mdx mice by IF methods [218,234,242,243]. In addition, although less studied, Sabatelli and colleagues have demonstrated an upregulation of collagen type IVα6 chain in the ECM of muscle biopsies of patients affected by DMD using IF methods [244].

#### 3.8.2. Fat Deposition

The process of adipogenesis during chronic muscle disease is still not well understood. As mentioned earlier, canonical FAPs (PDGFR-α^+^/Sca-1^+^) seem to be the main players, but other cell populations and factors released within the muscle niche regulate the process of ectopic fat accumulation [245]. Intramuscular fat is constituted by adipocytes, located between individual myofibres and intramyocellular lipids, stored inside the fibres as lipid droplets. The study of adipocytes has been hampered because the snap-freezing protocol generally used for skeletal muscle samples completely disrupts this cell morphology. Hence, while cryosectioning is key to preserve lipid droplets (lost in paraffin sections), the study of adipocytes must be carried out in previously fixed samples [38]. Consequently, to have an overall estimation of the lipid content of a muscle, the use of a classic lipophilic dye such as ORO in cryosections [246] is fast and the most generalised method [247]. This technique has been applied to understand the process of fat deposition in the context of muscle injuries and different dystrophies [221,248,249] and to test the efficiency of treatments in *mdx* mice [250]. Additionally, fluorescent dyes such as Bodipy are also useful tools to precisely quantify lipid droplets in a fibre type-specific manner [63]. Complementarily, to precisely recognise the adipocytes in prefixed samples, H&E staining or the use of specific antibodies to perilipin-1, the protein that coats lipid droplets in these cells is widely used. For full protocols, see: [38,249].

### 3.9. Calcification

Progression of the disease in patients with DMD is accompanied by an increase in ectopic calcification, especially in the heart and skeletal muscle fibres [251,252], a feature also found in different animal models [253,254,255,256].

As it has been shown that the development of these Ca^2+^ deposits also contributes to loss of force in skeletal and cardiac fibres [254], understanding the factors leading to their apparition and expansion is key to develop preventive therapies. Some lines of evidence suggest there is a link between the apparition of Ca^2+^ deposits and the general loss of Ca^2+^ homeostasis observed in dystrophic individuals [5,255,257]. In fact, calcification of dystrophic muscles is produced by the accumulation of hydroxyapatite (calcium phosphate) crystals [253,255] and increased concentrations of phosphorus in vivo [254] or in vitro [257] produces a parallel increase in Ca^2+^ deposits.

Although the molecular mechanisms leading to muscle calcification in muscles lacking dystrophin are still not clear, the process could be similar to the so-called “dystrophic calcification”, that is, the deposition of hydroxyapatite in soft tissues associated with previous damage/inflammation [258]. Importantly, even in healthy individuals, circulating levels of Ca^2+^ and phosphate are close to their solubility coefficient, so collagen or small Ca^2+^ deposits can act as nucleating factors that induce the deposition of crystals [259]. In patients with DMD and dystrophic animal models, increased ECM component deposition could act as a nucleating factor that, together with higher serum concentrations of Ca^2+^ [257] and phosphate [253], leads to skeletal muscle and heart calcification.

In line with this, levels of the profibrosis factor TGF-β and FAP cells directly correlate with extended calcification and higher severity of muscle pathogenesis in different *mdx* mice [260], linking fibrosis and the calcification process. Moreover, *mdx* mice fed a low-phosphorus diet showed no signs of calcification, and minimal muscle necrosis and inflammation in muscles of the lower limbs [254]. Finally, re-establishing Ca^2+^ homeostasis by targeting different Ca^2+^ handling proteins has been shown to ameliorate the DMD phenotype and improve muscle function (reviewed by [72]), demonstrating the contribution of Ca^2+^ overload to the process of mineralisation in dystrophic muscles.

Traditionally, Ca^2+^ and phosphorus deposits have been studied by Alizarin red [261] and von Kossa [262], respectively. These are commercially available kits that can be applied easily to both tissue sections and cell plates [221,263]. Moreover, some protocols permit the visualisation of calcified structures in whole-mount preparations [264]. Additionally, several recent studies have used computed tomography X-ray microscopy to obtain 3D images of in vivo limb muscles [254] or ex vivo whole-mount muscles [257], and assess their mineralised volume. Finally, electron back-scatter diffraction analysis not only permits location of mineralised areas as electron-dense spots, but also identifies the composition of the crystals [253,255].

### 3.10. Changes in Neuromuscular Junction

Like other mechanisms explained above, alteration in the DAPC due to the lack of dystrophin causes abnormalities in the presynaptic and postsynaptic regions of the NMJ. These structural abnormalities are accompanied by functional changes in neuromuscular transmission and nerve-evoked electromyography (reviewed by [265]).

Although there is some controversy in the literature [266,267,268], some studies clearly demonstrate that NMJ fragmentation and excessive nerve sprouting seen in animal models as well as patients with DMD are the result of continuous degeneration and regeneration of myofibres [269,270,271]. Furthermore, fragmentation of postsynaptic endplates observed in dystrophic muscle is a clear sign that the signalling cascades regulating NMJ morphology and plasticity after degeneration are also dysregulated. Specifically, dissociation of the DAPC alters the agrin signalling pathway, which is essential for acetylcholine receptor clustering (reviewed by [272]) and, thus, NMJ maturation [273]. Therefore, to date, several therapies targeting the main components of these cascades have been envisioned [274,275] and may yield promising results in patients suffering DMD.

While the study of NMJs with TEM has contributed immensely to the comprehension of the ultrastructural changes observed in muscle dystrophies [266,276,277], advances in fluorescence imaging had paved the way for discoveries contributing to the understanding of how NMJs develop, mature and change in healthy and diseased conditions [278,279,280]. In this regard, a methodological paper has recently been published explaining a protocol for the staining and subsequent analysis of NMJs in prefixed whole-mount muscle preparations [281]. With this method, motoneuron axons and nerve terminals are labelled with antibodies to the presynaptic neuronal marker neurofilament (NF) and synaptophysin (Syn), respectively, while postsynaptic acetylcholine receptors are labelled with the fluorescent-conjugated marker α-bungarotoxin (α-BTX). For fresh muscle samples, the reader is referred to [282]. Note that in both cases, to study the morphology of the NMJs, whole-mount preparations are recommended, as cross or longitudinal sections result in poor morphological assessment. Moreover, to obtain a perfect 3D reconstruction of the NMJ, Z-stacks not spaced less than 1 μm acquired with a confocal laser-scanning microscope are needed. The analysis of NMJ structure and morphology can be carried out manually with imaging software as explained in Pratt et al. [281], or semi-automatically with programmes such as the ones described on Table S1 [283]. Finally, the establishment of a robust, standardised methodology to assess the morphometric analysis of NMJs [284] has contributed immensely to the discovery of important species-specific differences in the structure and plasticity of NMJs [285].

### 3.11. Changes in Capillarisation

Ischemia and vascular dysfunction have been suggested as other important pathogenic mechanisms in DMD (Figure S1). Several lines of evidence show that blood perfusion is compromised in dystrophin-mutant muscles, leading to extensive areas of necrotic tissue [286,287,288]. The rationale behind the appearance of these ischemic regions is based on the one hand on the important role played by dystrophin in anchoring the protein neuronal nitric oxide synthase (nNOS) to the sarcolemma. This enzyme is essential for the production and release of nitric oxide (NO) to the vasculature, nurturing the myofibres. In healthy individuals, adequate blood perfusion during muscle contraction is ensured by NO release and consequent vasodilatation. However, in the absence of functional dystrophin, the enzyme nNOS is not close enough to the sarcolemma, there is not enough NO to vasodilate and active myofibres do not receive enough perfusion [12,289]. These events lead to functional ischemia in dystrophic muscles [290]. On the other hand, fibrotic tissue deposition could also alter blood perfusion to muscle cells. In patients with DMD, deposition of connective tissue increases the distance between myofibres and small-calibre vessels, leading to impaired gas exchange, reduced communication by soluble factors and compromised mechanical function [291]. Finally, another possible mechanism to explain vascular dysfunction in aged *mdx* mice could be linked to dystrophic SCs’ reduced capacity to promote angiogenesis during the regeneration process [292].

For all the above-mentioned reasons, to assess the benefits of therapeutic strategies designed to re-establish normal perfusion in myofibres [293], researchers should quantify different parameters related to capillary density and distribution within the muscle fibres (reviewed by [294]). Unfortunately, even though some programmes have been designed to automatically assess all these parameters (Muscle2View, Table S1), to the best of our knowledge, none of the papers analysing the role of capillary alterations in dystrophic muscles or possible therapies have described this mechanism in such detail.

Endothelial cells, pericytes and smooth muscle cells are the main components of different diameter blood vessels in tissues. These cells can be precisely identified with different well-characterised IHC and IF techniques [295]. Among them, and in the context of dystrophic muscles, the most used are biotinylate or fluorescein-labelled lectins such as *Ulex europaeus* agglutinin 1 (UEA 1, only for humans) [296] or isolectin B4 (IB4) from *Griffonia simplicifolia* [297], antibodies to the cluster of differentiation 31 (CD31) [12,298] for endothelial cells and to PDGFR-β, to neuron-glia antigen 2 (NG2) or to α-smooth muscle actin (α-SMA), for pericytes [12]. CD31, also recognised as platelet–endothelial cell adhesion molecule-1, is an adhesion molecule specifically expressed on endothelial cells, platelets and several immune cells [299]. Lectins (UEA 1 and IB4) are carbohydrate-binding proteins that bind to specific glycoproteins and glycolipids present on endothelial cells and may be perfused into the animal before sacrifice or incubated in the ex vivo tissue sample [10].

Finally, noteworthy capillary density and distribution play a critical role in oxygen supply to the myofibre and it is strongly linked to fibre phenotype. Hence, the complete assessment of changes in the capillary network of a specific muscle should be carried out in a fibre type-dependent manner. Currently, by applying IF techniques, several groups have successfully identified different fibre types simultaneously with capillaries and laminin. For specific protocols, see: [140,296,298].

### 3.12. Revertant Fibres and Detection of Dystrophin in Gene-Editing Therapies

As mentioned above, DMD is caused by different frameshift mutations leading to premature stop codons in the dystrophin gene, which abolish the synthesis of the functional protein. Surprisingly, in some patients with DMD [300] and in several animal models of the disease [301,302,303], single or clustered sporadic dystrophin-positive muscle fibres, called revertant fibres (RFs), are observed scattered among dystrophin-negative fibres. The mechanism behind the spontaneous emergence of these fibres is still a matter of strong debate [304]. They may arise from epigenetic alternative splicing resulting in skipping exon(s) flanking the mutated exon, a process that would eliminate the mutation from the dystrophin transcript, and therefore, would restore the open reading frame of the shortened transcript [305,306,307]. Or alternatively, they are produced as the result of a second spontaneous mutation that corrects the original mutation in the DNA [301,308,309].

The clustered appearance of RFs in the adult and increased number with age [306,310] have led to the postulation of a “clonal expansion model”. According to this hypothesis, alternative splicing or second mutations would spontaneously occur in some myogenic progenitors early in muscle development, and later, these revertant precursors will expand during the cycles of degeneration/regeneration occurring in dystrophic mutants. Since these dystrophin^+^ RFs are more resistant to degeneration, their number will increase as the individual ages [311]. Independently of the mechanism responsible for their appearance and expansion, results demonstrating co-restoration of sarcolemmal DAPC proteins in these RFs [306] suggested that these fibres are less vulnerable to damage and have opened a window for the development of gene-editing experimental therapies [312,313,314].

Therefore, histological assessment of the dystrophin protein in dystrophic muscles is key, not only to further study RFs [304], but also to quantify the long-term effect and efficacy of exon-skipping therapies [315]. Hence, depending on the dystrophic model chosen, its specific mutation and the exons at which the genetic therapy is directed, researchers must carefully choose in between a broad spectrum of dystrophin monoclonal antibodies, each of which is designed to recognise specific dystrophin epitopes [316,317].

## 4. Conclusions

As our understanding of the molecular mechanisms leading to DMD increases, the search for new therapies combining drugs and genetic approaches evolves. To test these treatments and prove their safety and efficacy before clinical implementation, their application in animal models of the disease is critical. Moreover, objective assessment of the benefits of these experimental approaches must rely on validated methods of administration and the establishment of standardised analytical protocols. In the context of DMD, the degenerative/regenerative phase desynchronisation between different muscles and even within the same muscle makes ex vivo histopathological study a reliable tool to assess the overall condition of muscle fibres, as it yields important locational information that other valuable techniques such as WB or RT-qPCR cannot provide.

As microscopy technology progresses and pharmacological companies expand their catalogues with innumerable primary antibodies for IH evaluation, the researcher needs clear, standardised and reliable protocols to objectively evaluate the beneficial effects a therapy could exert on the progression of the disease. Here, we presented a summary of the most commonly used histochemical and immunohistological techniques, we explained the rationale behind the use of certain markers and we cited reliable protocols to guide the researcher. Importantly, the paper stresses that some of the methods used in the past have been substituted with either easier or more reliable protocols and citations are provided.

The main limitation of this article is the impossibility of addressing all possible markers and stains ever used in the field of dystrophinopathies. Nevertheless, we provide markers of degeneration for necrotic/pyroptotic fibres, markers of inflammation and inflammatory cells, means to evaluate oxidative stress and mitochondrial function, protocols to assess the regenerative process including markers for SC proliferation and differentiation, markers of myofibre maturation and branching and markers for FAPs, together with methods to evaluate fibrosis, fat accumulation and calcification, capillarisation and, finally, NMJ reorganisation. Furthermore, we also explain mechanisms of analysis and how to interpret the results of some of these histological tests.

## Supplementary Materials

The following supporting information can be downloaded at: https://www.mdpi.com/article/10.3390/ijms232416080/s1. References [318,319,320,321,322,323,324,325,326,327,328,329,330,331,332,333] are cited in the supplementary materials.

## Abbreviations

**8-OHdG**	8-hydroxy-2′-deoxyguanosine
**α-BTX**	alpha bungarotoxin
**α-SMA**	alpha smooth muscle actin
**ALP**	alkaline phospatase
**APC**	antigen-presenting cells
**CCR**	C-C chemokine receptor
**CD**	cluster of differentiation
**CNF**	centrally nucleated fibres
**COX**	cytochrome C oxidase
**CSA**	cross sectional area
**CTGF**	connective tissue growth factor
**DAMPs**	damage-associated molecular patterns
**DAPC**	dystrophin-associated protein complex
**DAPI**	4′,6-diamidino-2-phenylindole
**DHE**	dihydroethidium
**DMD**	Duchenne muscular dystrophy
**EBD**	Evan’s blue dye
**ECM**	extracellular matrix
**emb-MyHC**	embryonic myosin heavy chain
**FAPs**	fibro-adipogenic progenitors
**FOXP3**	Forkhead box protein 3
**GT**	Gömöri trichrome
**H&E**	haemotoxylin and eosin
**HNE**	4-hydroxynonenal
**IB4**	isolectin B4 from *Griffonia Simplicifolia*
**IFNγ**	interferon gamma
**Ig**	immunoglobulin
**IH**	immunohistology/immunohistological
**IL-10**	interleukin 10
**IL-1β**	interleukin 1 beta
**IL-4**	interleukin 4
**IL-6**	interleukin 6
**iNOS**	inducible nitric oxide synthase
**M’sT**	Masson’s trichrome
**MF’sD**	minimum Feret’s diameter
**MPO**	myeloperoxidase
**MyHC**	myosin heavy chain
**MyoD**	myoblast determination protein 1
**Myog.**	myogenin
**NF**	neurofilament
**NG2**	neuron-glial antigen 2
**NMJ**	neuromuscular junction
**NO**	nitric oxide
**nNOS**	neuronal nitric oxide synthase
**ORO**	Oil red O
**Pax7**	paired box protein 7
**PDGFR-α**	platelet-derived growth factor receptor-alpha
**PDGFR-β**	platelet-derived growth factor receptor-beta
**PR**	Picrosirius red
**RF**	revertant fibre
**ROS**	reactive oxygen species
**RT-qPCR**	real-time quantitative polymerase chain reaction
**SC**	satellite cell
**SDH**	succinate dehydrogenase
**SR**	sarcoplasmic reticulum
**Syn**	synaptophysin
**TEM**	transmission electron microscopy
**TGF-β**	transforming growth factor-beta
**TCF4**	transcription factor 4
**Treg**	T regulatory cell
**TNFα**	tumour necrosis factor alpha
**UEA 1**	*Ulex europaeus* agglutinin 1
**WB**	Western blotting
**WGA**	wheat germ agglutinin
**WT**	wild type
