# Supplementary material for: Histological Methods to Assess Skeletal Muscle Degeneration and Regeneration in Duchenne Muscular Dystrophy
Source: Int J Mol Sci. 2022 Dec 16;23(24):16080. doi: 10.3390/ijms232416080 (PMC9786356; doi:10.3390/ijms232416080)
Supplement: Supplementary file 1 [file ijms-23-16080-s001.zip › Figure S1 - Dubuisson et al., IJMS - reviewed by authors.pdf]

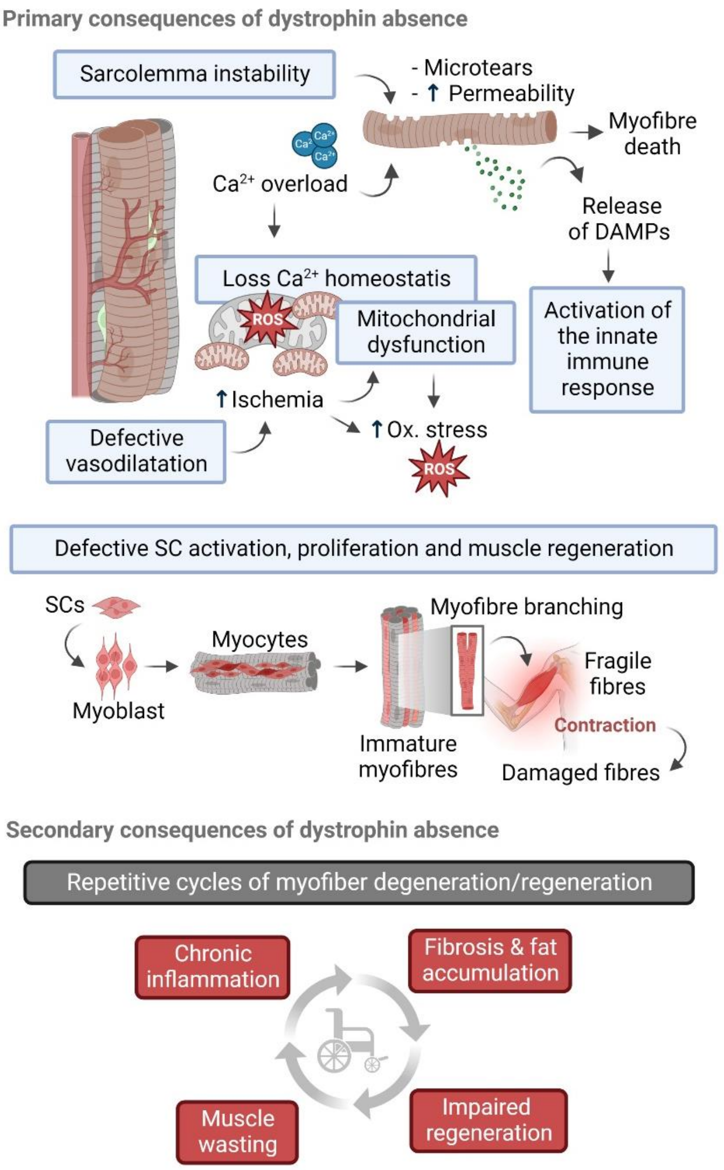

Figure S1. Main molecular events leading to functional impairment in dystrophic muscle. Several non-exclusive molecular events (LIGH BLUE RECTANGLES) could be responsible for the physiopathology in dystrophic myopathies. Lack of dystrophin in the sarcolemma of skeletal muscle fibres affects the assembling of the dystroglycan-associated protein complex (DAPC) and results in sarcolemmal instability during contraction. This leads to Ca^{2+} cell overload, a process that is exacerbated by a parallel dysfunction of Ca^{2+}-handling proteins, resulting in the loss of Ca^{2+} homeostasis. This impairs mitochondria resulting in increased oxidative stress. In parallel, lack of dystrophin also contributes to defective vasodilatation since the hormone arginine, produced by the mislocated DAPC enzyme nNOS, cannot reach the capillaries. Therefore, impaired gas exchange produces ischemia, damaging mitochondria, and contributing to increased oxidative stress. The above-mentioned events cause the release of damage-associated molecular patterns (DAMPs) from myofibres and activate the innate immune response. In healthy muscles, immune-derived cytokines activate quiescent SCs that proliferate and differentiate to form myocytes that contribute to the regeneration of damaged fibres. Lack of dystrophin in SCs also affects their correct activation, proliferation, and differentiation. Hence, new myotubes bind to dystrophic myofibres and produce branched fibres that are more vulnerable to contraction. The sarcolemma of these branched fibres will be damaged upon contraction and the degenerative process will start all over. Thus, as a consequence of all the primary causes produced by the lack of dystrophin, a myriad of secondary events (RED RECTANGLES) will contribute to general impaired muscle function in Duchenne muscular dystrophy patients. Among them, chronic inflammation, impaired muscle regeneration, fibrosis and fat accumulation and resultant muscle wasting, are the most important.
