# Supplementary material for: Histological Methods to Assess Skeletal Muscle Degeneration and Regeneration in Duchenne Muscular Dystrophy
Source: Int J Mol Sci. 2022 Dec 16;23(24):16080. doi: 10.3390/ijms232416080 (PMC9786356; doi:10.3390/ijms232416080)
Supplement: Supplementary file 1 [file ijms-23-16080-s001.zip › Table S1 - Programs Skeletal Muscle Analysis - reviewed by authors.pdf]

Table S1. Main characteristics of available programs and algorithms to assess skeletal muscle morphometrical/regenerative features.

**Characteristic**	**SMASH**	**MyoVision**	**MuscleJ**	**NO NAME**	**Open-CSAM**	**CellProfiler + Muscle Analyzer**	**MacroIMRB**	**musclefibertyiping**	**MyoSoft**	**FLIKA + QuantiMus**	**NO NAME**	**CellProfiler + Muscle2 View**	**Cellpose + LabelsToROIS**	**MyoSight**
**Reference**	[1]	[2]	[3]	[4,5]	[6]	[7]	[8]	[9,10]	[11]	[12]	[13]	[14]	[15,16]	[17]
**# of cites WOS** *(Last updated 21/11/2022)*	158	94	52	28	26	21	20	7 / 18	16	12	12	11	11	6
**Compatibility** *(Several: 1 point)*	PC	PC	PC/Mac/Linux	PC/Mac/Linux	PC/Mac/Linux	PC/Mac/Linux	PC/Mac/Linux	PC/Mac/Linux	PC/Mac/Linux	PC/Mac/Linux	PC/Mac/Linux	PC/Mac/Linux	PC/Mac/Linux	PC/Mac/Linux
**License / Availability** *(Free: 1 point)*	Free [a]	Free [a]	Free	Free	Free	Free	Free	Free	Free	Free	Free	Free	Free	Free
**Programming Language // Platform**	MATLAB	MATLAB	Python // FIJI	Algth. *(not associated with a platform)*	Python // FIJI	Python *(pipeline)*	Python // FIJI	Python // FIJI	Python // FIJI	Python	Algth. *(implemented in MATLAB)*	Python *(pipeline)*	Jython // FIJI	Python // FIJI
**Image type** *(Diff. formats: 1 point)*	Bmp, jpg, png, tif, TIFF	Png, jpg, tif, jpeg, TIFF	OF, TIFF 16 bits	Not mentioned	TIFF [b], jpeg, png	All standard image formats	TIFF 16 bits	jpeg	OF, TIFF 8 or 16 bits	TIFF 8 bits	Not mentioned	TIFF	OF, Jpg, gif, png, TIFF	OF, TIFF, jpeg
**Processing whole-muscle cross sections** *(1 point)*	+	−	+	−	+	+	+	+	+	+	+	−	+	−
**Auto/semi-automated**	SA	A	A	−	SA	A	A	A	A	SA	−	A	SA	SA
**Manual corrections** *(1 point)*	+	−	+ [c]	?	+	−	−	−	+ [c]	+	?	−	+	+
**Fiber area/perimeter** *(1 point)*	+	+	+	+	+	+	+	−	+	+	+	+	+	+
**Min. & Max Feret's Diameter** *(1 point)*	+	+	+	−	+	+	+	−	+	+	+	+	+	+
**Identifies fiber-types** *(1 point)*	+	+	+	+	−	−	+	+	+	+	−	+	−	+
**Identifies hybrid fibers** *(1 point)*	− [d]	−	−	+	−	−	−	−	+	−	−	−	−	+
**Identifies central nuclei** *(1 point)*	+	−	+	+	−	+	−	−	−	+	−	+	−	+

**# central nuclei on each fiber** *(1 point)*	–	–	+	–	–	–	–	–	–	–	–	+	–	+
**Identifies perinuclei** *(1 point)*	–	+	+	+	–	–	–	–	–	–	–	+	–	+
**Identifies satellite cells** *(1 point)*	–	–	+	–	–	–	–	–	–	–	–	– [e]	–	–
**Identifies blood vessels** *(1 point)*	+	–	+	–	–	–	–	–	–	–	–	+ [f]	–	–
**Processing Z-stacks** *(1 point)*	–	–	+	–	–	–	–	–	–	–	–	–	–	–
**Result files automatically generated** *(1 point)*	+	+	+	–	– [g]	+	+	–	+	+	–	+	–	+
**Batch analysis of images** *(1 point)*	–	+	+	–	–	+	+	+	–	–	–	+	+	+ [h]
**Format of generated results**	Excel	Excel	csv tables (Excel)	–	txt or csv tables (Excel)	csv tables (Excel)	csv tables (Excel)	csv tables (Excel)	csv tables (Excel)	Excel	–	Excel	csv tables (Excel)	txt
**Visual output of analyses performed** *(1 point)*	+	–	+	–	–	+	+	–	+	–	?	–	+	+
**Written or video tutorials** *(1 point)*	+	+	+	–	+	+	+	+	+	+	–	+ [i]	+	+
**Programming skills requierd** *(No: 1 point)*	No	No	No	Yes	No	No	No	Yes [j]	No	No	Yes	No	Yes [k]	No
**Link to turorials**	SMASH	MyoVision	MuscleJ	–	Open-CSAM	CellProfiler	MacroIMRB	MuscleFiberTyping	MyoSoft	QuantiMus	–	CellProfiler	Cellpose LabelsToROIs	MyoSight
**SCORE GIVEN** *(Out of 20 point)*	13	10	19	7	9	12	11	6	13	11	5	13	10	16

Comparisons of a selection of 10 freely available muscle-related image analysis programs. The selection was made based on the number of cites (sources: Web of Science, updated November 21st 2022). This comparison includes the basic characteristics of each program, type of images needed, batch processing of images, format of the results obtained, the availability of tutorials and if any programming skills are required to use it. It is important to stress that to optimize the results, even on the programs that are fully automated and require little or none programming skills, some machine-user interaction and optimization are needed to adapt the settings to the quality and characteristics of the user's images and needs. A final score of up to 20 points was assigned to each program based on the number of positive items or benefits achieved. Both Muscle Analyzer and Muscle2View are not programs *per se*, they are a

set of pipelines or image-processing modules to be run on the imaging processing software CellProfiler. **Abbreviations;** Algth: Algorithms, OF: original format images obtained from the acquisition system, A: Automated, SA: Semi-Automated. **Superindex:** (a) Runs under MATLAB Runtime Compiler. (b) Tiff is the preferential format, however, other formats like jpeg and png can be used. (c) shown on MuscleJ and Myosoft indicate that some corrections can be made after the results are obtained by uploading the corresponding ROI.zip file and deleting/adding new ROIs. The user must be familiar with ImageJ/Fiji environment. (d) shown on SMASH indicates that the program does not recognize hybrid fibers, however, fibers showing medium staining intensity for any marker, could be assessed as a hybrid fiber, but the user must manually check its staining for other markers to assign it to an hybrid group. (e) shown on CellProfiler+Muscle2View indicates that this program could quantify satellite cells but some adjustments in the pipeline are required. (f) shown on CellProfiler+Muscle2View indicates that this program makes an extensive analysis of the fiber type-specific capillary network, including the capillary contacts and sharing factor (no. of fibers sharing one capillary) for each individual fiber. (g) shown on OpenCSAM indicates a table show the results, but the user must manually save the table. (h) shown on MyoSight indicates that the program permits the analysis of several images, but each time, the user must select the image to be analyzed and the folder to save the results. (i) shown on CellProfiler+Muscle2View indicates that there is only a tutorial for CellProfiler, but not for the Muscle2View pipeline. (j) shown on musclefibertyping indicates that minor modifications of the macro are needed to change the directory where the pictures are stored. (k) shown on Cellpose+LabelsToROIs indicates that high computer skills are required for the installation and use of Cellpose.

References

1. Smith, L.R.; Barton, E.R. SMASH—Semi-Automatic Muscle Analysis Using Segmentation of Histology: A MATLAB Application. *Skelet Muscle* **2014**, *4*, 21. https://doi.org/10.1186/2044-5040-4-21.
2. Wen, Y.; Murach, K.A.; Vechetti, I.J.; Fry, C.S.; Vickery, C.; Peterson, C.A.; McCarthy, J.J.; Kenneth Campbell, X.S.; Jr, V.I. INNOVATIVE METHODOLOGY MyoVision: Software for Automated High-Content Analysis of Skeletal Muscle Immunohistochemistry. *J. Appl. Physiol.* **2018**, *124*, 40–51. https://doi.org/10.1152/japplphysiol.00762.2017.
3. Mayeuf-Louchart, A.; Hardy, D.; Thorel, Q.; Roux, P.; Gueniot, L.; Briand, D.; Mazeraud, A.; Bouglé, A.; Shorte, S.L.; Staels, B.; et al. MuscleJ: A High-Content Analysis Method to Study Skeletal Muscle with a New Fiji Tool. *Skelet Muscle* **2018**, *8*, 25. https://doi.org/10.1186/s13395-018-0171-0.
4. Mula, J.; Lee, J.D.; Liu, F.; Yang, L.; Peterson, C.A. Automated Image Analysis of Skeletal Muscle Fiber Cross-Sectional Area. *J. Appl. Physiol.* **2013**, *114*, 148–155. https://doi.org/10.1152/japplphysiol.01022.2012.-Morphological.
5. Liu, F.; Fry, C.S.; Mula, J.; Jackson, J.R.; Lee, J.D.; Peterson, C.A.; Yang, L. Automated Fiber-Type-Specific Cross-Sectional Area Assessment and Myonuclei Counting in Skeletal Muscle. *J. Appl. Physiol.* **2013**, *115*, 1714–1724. https://doi.org/10.1152/japplphysiol.00848.2013.-Skele.
6. Desgeorges, T.; Liot, S.; Lyon, S.; Bouvière, J.; Kemmel, A.; Trignol, A.; Rousseau, D.; Chapuis, B.; Gondin, J.; Mounier, R.; et al. Open-CSAM, a New Tool for Semi-Automated Analysis of Myofiber Cross-Sectional Area in Regenerating Adult Skeletal Muscle. *Skelet Muscle* **2019**, *9*, 2. https://doi.org/10.1186/s13395-018-0186-6.
7. Lau, Y.S.; Xu, L.; Gao, Y.; Han, R. Automated Muscle Histopathology Analysis Using CellProfiler. *Skelet Muscle* **2018**, *8*, 32. https://doi.org/10.1186/s13395-018-0178-6.
8. Reyes-Fernandez, P.C.; Periou, B.; Decrouy, X.; Relaix, F.; Authier, F.J. Automated Image-Analysis Method for the Quantification of Fiber Morphometry and Fiber Type Population in Human Skeletal Muscle. *Skelet Muscle* **2019**, *9*, 15. https://doi.org/10.1186/s13395-019-0200-7.
9. Bergmeister, K.D.; Gröger, M.; Aman, M.; Willensdorfer, A.; Manzano-Szalai, K.; Salminger, S.; Aszmann, O.C. A Rapid Automated Protocol for Muscle Fiber Population Analysis in Rat Muscle Cross Sections Using Myosin Heavy Chain Immunohistochemistry. *J. Vis. Exp.* **2017**, *2017*, e55441. https://doi.org/10.3791/55441.

10. Bergmeister, K.D.; Gröger, M.; Aman, M.; Willensdorfer, A.; Manzano-Szalai, K.; Salminger, S.; Aszmann, O.C. Automated Muscle Fiber Type Population Analysis with ImageJ of Whole Rat Muscles Using Rapid Myosin Heavy Chain Immunohistochemistry. *Muscle Nerve* **2016**, *54*, 292–299. https://doi.org/10.1002/mus.25033.
11. Encarnacion-Rivera, L.; Foltz, S.; Hartzell, H.C.; Choo, H. Myosoft: An Automated Muscle Histology Analysis Tool Using Machine Learning Algorithm Utilizing FIJI/ImageJ Software. *PLoS ONE* **2020**, *15*, e0229041. https://doi.org/10.1371/journal.pone.0229041.
12. Kastenschmidt, J.M.; Ellefsen, K.L.; Mannaa, A.H.; Giebel, J.J.; Yahia, R.; Ayer, R.E.; Pham, P.; Rios, R.; Vetrone, S.A.; Mozaffar, T.; et al. QuantiMus: A Machine Learning-Based Approach for High Precision Analysis of Skeletal Muscle Morphology. *Front. Physiol.* **2019**, *10*, 1416. https://doi.org/10.3389/fphys.2019.01416.
13. Miazaki, M.; Viana, M.P.; Yang, Z.; Comin, C.H.; Wang, Y.; da F Costa, L.; Xu, X. Automated High-Content Morphological Analysis of Muscle Fiber Histology. *Comput. Biol. Med.* **2015**, *63*, 28–35. https://doi.org/10.1016/j.compbiomed.2015.04.020.
14. Sanz, G.; Manuel Martínez-Aranda, L.; Tesch, P.A.; Fernandez-Gonzalo, R.; Tommy, X.; Lundberg, R. INNOVATIVE METHODOLOGY Muscle2View, a CellProfiler Pipeline for Detection of the Capillary-to-Muscle Fiber Interface and High-Content Quantification of Fiber Type-Specific Histology. *J. Appl. Physiol.* **2019**, *127*, 1698–1709. https://doi.org/10.1152/japplphysiol.
15. Stringer, C.; Wang, T.; Michaelos, M.; Pachitariu, M. Cellpose: A Generalist Algorithm for Cellular Segmentation. *Nat. Methods* **2021**, *18*, 100–106. https://doi.org/10.1038/s41592-020-01018-x.
16. Waisman, A.; Norris, A.M.; Elías Costa, M.; Kopinke, D. Automatic and Unbiased Segmentation and Quantification of Myofibers in Skeletal Muscle. *Sci. Rep.* **2021**, *11*, 11793. https://doi.org/10.1038/s41598-021-91191-6.
17. Babcock, L.W.; Hanna, A.D.; Agha, N.H.; Hamilton, S.L. MyoSight—Semi-Automated Image Analysis of Skeletal Muscle Cross Sections. *Skelet Muscle* **2020**, *10*, 33. https://doi.org/10.1186/s13395-020-00250-5.
